# The Effects of Baicalin in Combination with Cefotaxime on the Biofilm and Metabolic Reprogramming of Multidrug-Resistant *Pseudomonas aeruginosa*

**DOI:** 10.3390/biom16040598

**Published:** 2026-04-17

**Authors:** Xin Meng, Chao Ning, Xinyu Lu, Mengna Kang, Yuxuan Yang, Zhiyun Yu, Yu Wang, Yantong Sun, Haiyong Guo

**Affiliations:** 1 College of Life Science, Jilin Normal University, Siping 136000, China; 2 School of Pharmaceutical Sciences, Jilin University, Changchun 130021, China

**Keywords:** baicalin, multidrug-resistant *Pseudomonas aeruginosa*, cell wall, biofilm, metabolism

## Abstract

Baicalin, a natural plant-derived compound, holds promise in addressing clinical bacterial resistance when combined with antibiotics. This study evaluated the antibacterial activity of the combination of baicalin and cefotaxime and explored its mechanism of action on the cell wall and biofilm of multidrug-resistant *Pseudomonas aeruginosa* (MRPA). The results showed that the combination of baicalin and cefotaxime exerted a synergistic inhibitory effect on the growth of MRPA, with a fractional inhibitory concentration index (FICI) of 0.28. Mechanistically, compared with cefotaxime alone, the combination of baicalin and cefotaxime enhanced the permeability of the cell membrane and cell wall of MRPA, thereby increasing cell damage. It also exhibited stronger antibiofilm activity by inhibiting numerous virulence factors (pyocyanin, elastase, lectin), reducing cellular metabolic activity, and downregulating the expression of biofilm genes (*pslA*, *pelA*, *algD*) and quorum-sensing genes (*lasl*, *lasR*, *rhll*, *rhlR, pqsA, pqsR*). The molecular docking results revealed that baicalin could stably bind to wbpE, LasR, and RhlR. Therefore, this interaction may indirectly influence the processes related to antibiotic resistance and biofilm formation in bacterial cells. Metabolomic analysis revealed that the combination of baicalin and cefotaxime upregulated 863 metabolites and downregulated 587 metabolites. These metabolites mainly included amino acids, lipids, nucleotides, carbohydrates, and secondary metabolites. The combination primarily enriched key pathways such as amino acid metabolism, lipid metabolism (sphingolipid metabolism) and secondary metabolite biosynthesis. Through these pathways, it triggers significant metabolic reprogramming, thereby interfering with the supply of cell wall synthesis precursors, membrane structural stability, and the generation of biomembrane matrix. Ultimately, it synergistically enhances the effects of cell wall damage and biomembrane inhibition. In conclusion, this study confirms that the combination of baicalin and cefotaxime exerts significant synergistic antibacterial activity against MRPA. It also reveals the mechanism of action of the combination on the cell wall and biofilm of MRPA at the metabolic level, providing theoretical support for the development of novel strategies to combat MRPA.

## 1. Introduction

As a Gram-negative bacterium, *Pseudomonas aeruginosa* can extensively infect both animal and plant hosts [1]. It is responsible for 10–15% of common nosocomial infections in clinical settings [2], and notably, it acts as a major pathogen causing chronic pulmonary infections in patients with cystic fibrosis [3]. In recent years, the emergence of multidrug-resistant *Pseudomonas aeruginosa* (MRPA) has posed a serious threat to public health. Studies have shown that the resistance mechanisms of *Pseudomonas aeruginosa* to cephalosporin antibiotics are complex and diverse, involving the synergistic interaction of multiple key links [4]. As β-lactam antibiotics, cephalosporins exert their antibacterial effect by binding to bacterial penicillin-binding proteins (PBPs) to inhibit cell wall synthesis. However, this bacterium can inactivate cephalosporins by producing various β-lactamases that hydrolyze the β-lactam ring, which is one of its major resistance mechanisms [4]. Meanwhile, reduced expression or structural mutations of outer membrane porins lead to decreased outer membrane permeability, thereby impairing drug uptake efficiency [5,6]. Additionally, biofilms formed during chronic infections can hinder drug penetration through physical barrier effects and reduce bacterial metabolic activity, ultimately resulting in decreased bacterial sensitivity to drugs [7].

A biofilm is a synergistic community structure formed by bacteria enclosed in self-secreted extracellular polymeric substances (EPSs), which can protect themselves from elimination by the host immune system and adverse external environmental influences [8]. Extracellular polymeric substances consist of exopolysaccharides, proteins, extracellular DNA (eDNA), and lipids [9,10], among which exopolysaccharides are the main component [11]. The three polysaccharides (Psl, Pel, and alginate) synthesized by *Pseudomonas aeruginosa* each have unique functions: Psl maintains biofilm structure and promotes microcolony formation [12]; Pel enhances biofilm resistance and stability [13,14]; and alginate protects cells and strengthens adhesion ability [15,16]. Collectively, these polysaccharides impart the biofilm matrix with its characteristic physiological properties.

*Pseudomonas aeruginosa* secretes various virulence factors, such as adhesion proteins, elastase, and pyocyanin, which are involved in metabolic regulation, motility adjustment, and maintenance of biofilm stability, thereby enhancing its survival and pathogenic capabilities [17,18]. The quorum-sensing (QS) system regulates the production of virulence factors through signal molecules of the las, rhl, and PQS systems, while coordinating biofilm formation and function [19,20,21,22]. In summary, *Pseudomonas aeruginosa* constructs a complex network of drug resistance and pathogenicity by regulating cell membrane permeability, promoting biofilm formation, synthesizing virulence factors, and activating the quorum-sensing (QS) system, which underscores the urgency of developing alternative therapeutic strategies. Baicalin is a flavonoid compound extracted from the plant *Scutellaria baicalensis* [23]. It has been proven to have various pharmacological activities [23]. In particular, its antibacterial properties and the fact that it is unlikely to cause drug resistance make it promising as an antibiotic adjuvant to address the problem of bacterial resistance in clinical settings [23]. Existing studies have shown that the combination of baicalin and antibiotics can alleviate bacterial resistance, while reducing the concentration of antibiotics used and their toxic side effects [24]. However, the mechanism underlying their synergistic effect remains to be explored. This study aims to evaluate the antibacterial activity of baicalin combined with cefotaxime against MRPA, and to investigate its antibacterial mechanism on MRPA from aspects such as cell membrane and cell wall permeability, biofilm composition and metabolic activity, virulence factors, quorum sensing gene expression, and metabolomics.

## 2. Materials and Methods

### 2.1. Bacterial Strains, Cell Cultures, and Chemicals

*Pseudomonas aeruginosa* (PAO1) was purchased from Shanghai Miaoling Biotechnology Co., Ltd. (Shanghai, China), and multidrug-resistant *Pseudomonas aeruginosa* was obtained from Beijing Bio-Emperor Biotechnology Co., Ltd. (Beijing, China). Both strains were aerobically cultured on LB agar at 37 °C for 24 h. Single colonies were picked and inoculated into 5 mL of LB broth, followed by incubation at 37 °C for 8 h to reach the logarithmic phase (1 × 10^8^ CFU/mL). In the experiment, sterile water was used as the negative control, and gentamicin (GM) served as the positive control. GM was purchased from Shanghai Aladdin Biochemical Technology Co., Ltd. (Shanghai, China). Cefotaxime (CTX) was obtained from MedChemExpress (MCE, Monmouth Junction, NJ, USA). Baicalin (BA, HPLC purity ≥ 98%), Congo red, and crystal violet were purchased from Beijing Solarbio Science & Technology Co., Ltd. (Beijing, China). The BCA protein assay kit was obtained from Nanjing Best Biotechnology Co., Ltd. (Nanjing, China). The alkaline phosphatase (AKP) assay kit was purchased from Nanjing Jiancheng Bioengineering Institute (Nanjing, China). Total RNA extraction reagent (Trizol) and qPCR primers were obtained from Shanghai Sangon Biotech Co., Ltd. (Shanghai, China). MTT was purchased from Beijing Land Bridge Technology Co., Ltd. (Beijing, China), and phenol was obtained from Shanghai Yueteng Biotechnology Co., Ltd. (Shanghai, China). The microbial lectin ELISA detection kit was purchased from Shanghai Jining Biological Technology Co., Ltd. (Shanghai, China).

### 2.2. Minimum Inhibitory Concentration (MIC) Assay

Follow the method of Meng et al. [25]. For all the drugs, a two-fold dilution was performed. Among them, the concentration ranges of BA, CTX and GM were 2500 to 15,000 µg/mL, 3.9–62.5 µg/mL and 0.39–6.25 µg/mL respectively.

### 2.3. Checkerboard Dilution Assay

BA and CTX were subjected to two-fold serial dilution along the *x*-axis and *y*-axis of a 96-well plate, respectively, starting from their individual minimum inhibitory concentrations, with 6 concentration gradients established for each compound. Subsequently, the bacterial suspension was added according to the method of Meng et al. [25] and absorbance was measured at 600 nm. The fractional inhibitory concentration index (FICI) was calculated according to the method described by Tyagi et al. [26].

### 2.4. Growth Curve Determination

Bacterial suspension (2 × 10^5^ CFU/mL) was mixed with equal volumes of test agents, including BA, CTX, and the combination of BA and CTX. Subsequently, the effect of drug combination on bacterial growth was detected using the method proposed by Meng et al. [25], and the growth curve was plotted.

### 2.5. Bactericidal Kinetics Assay

The bacterial suspension was diluted to 2 × 10^5^ CFU/mL and incubated with different drugs (BA, CTX, and BA + CTX) for different time intervals. Then, after appropriate dilution, it was spread onto an LB agar plate and incubated overnight before counting. Bactericidal kinetic curves were constructed based on the results.

### 2.6. Scanning Electron Microscopy (SEM) Assay

According to the method of Meng et al. [25], the bacterial suspension was incubated with different drugs (BA, CTX, and BA + CTX) for 1 h. Subsequently, the bacterial cells were collected, washed, and fixed. Then, a series of concentrations of ethanol were used for gradient elution, and finally, they were subjected to freeze-drying. The samples were then subjected to vacuum sputter coating with a ~5 nm gold/palladium layer before being observed under a scanning electron microscope (Hitachi High-Tech Corporation, Tokyo, Japan).

### 2.7. Intracellular Protein Leakage Assay

The overnight-cultured bacteria were washed with PBS and diluted to 1 × 10^6^ CFU/mL. The bacteria were incubated with different drugs (BA, CTX, and BA + CTX) in a low-temperature shaking (low-speed) environment for 6 h. The supernatant was collected by centrifugation and the protein content in the bacterial extracellular matrix after drug treatment was detected using the BCA protein assay kit.

### 2.8. Alkaline Phosphatase (AKP) Activity Assay

According to the method of Chen et al. [27], a bacterial suspension of 1 × 10^6^ CFU/mL was prepared and incubated with different drugs for 2 h. The supernatant was then collected by centrifugation and AKP activity was detected using the AKP activity detection kit.

### 2.9. Molecular Docking

The two-dimensional chemical configuration of the bioactive compound BA was acquired from PubChem, with its three-dimensional conformation subsequently generated through Chem3D software. Crystallographic data for *Pseudomonas aeruginosa* WbpE transaminase (PDB entry: 3NU7), LasR transcriptional regulator (PDB entry: 6D6A), and RhlR protein (PDB entry: 4Y15) were obtained from the Protein Data Bank. Molecular preparation of both ligand and target proteins was conducted using AutoDock Tools version 1.5.6 before performing docking simulations. In this investigation, BA functioned as the small molecule ligand, whereas WbpE transaminase, LasR, and RhlR proteins were designated as macromolecular targets. Structural refinement was achieved through energy minimization employing the Amber14 force field, implemented in a sequential optimization protocol: initial structural adjustment involved 1000 iterations of steepest descent algorithm, succeeded by 5000 cycles of conjugate gradient optimization for final structural refinement. The resulting energy-minimized structure served as the basis for all subsequent computational analyses.

### 2.10. Antibiofilm Activity Assay

To assess the suppression of biofilm development, microbial suspensions containing 1 × 10^6^ CFU/mL were exposed to experimental compounds for 24 h. Following this exposure, unattached microorganisms were eliminated through three successive phosphate-buffered saline washes. The remaining biofilms were immobilized using methanol treatment for 25 min. These immobilized structures were then subjected to coloration with a 0.1% crystal violet solution, with subsequent removal of unbound dye through extensive rinsing. The attached stain was subsequently dissolved using 95% ethanol solution, and optical density readings at 590 nm wavelength were recorded to quantify the compounds’ capacity to hinder biofilm formation.

In experiments examining the degradation of established biofilms, microbial communities were allowed to mature for 24 h prior to treatment. These developed biofilms were then exposed to the test substances, after which the identical analytical procedure was implemented. This included phosphate-buffered saline rinsing, methanol-based fixation, crystal violet application, ethanol-mediated dissolution of the dye complex, and spectrophotometric analysis at 590 nm to determine the compounds’ efficacy in disrupting pre-existing biofilm structures.

### 2.11. Confocal Laser Scanning Microscopy (CLSM) Analysis

The effect of the combination of BA and CTX on the biomass of MRPA biofilms was observed using a confocal laser scanning microscope. Bacterial suspension (1 × 10^6^ CFU/mL) was co-incubated with test agents overnight in confocal culture dishes. Following incubation, unattached cells were eliminated through phosphate-buffered saline rinses. Cellular viability assessment was conducted using the LIVE/DEAD BacLight™ Bacterial Viability Kit (Invitrogen, Life Technologies, Shanghai, China), with SYTO9 fluorescent dye marking viable microorganisms and propidium iodide identifying nonviable cells. Fluorescence imaging was subsequently performed using a confocal microscope (Olympus Corporation, Tokyo, Japan).

### 2.12. Extracellular Polymeric Substance (EPS) Detection Assay

The bacterial culture was mixed with experimental compounds and incubated at 37 °C with continuous agitation for 36 h. Following incubation, the mixture underwent centrifugation at 3500× *g* in a refrigerated environment for 20 min to separate the supernatant. The remaining bacterial cells were then reconstituted in 2 mL of EDTA solution, vigorously mixed using a vortex device, and subjected to another centrifugation step to obtain additional supernatant. Both supernatant fractions were pooled together, after which chilled absolute ethanol (2.2 times the volume) was introduced to the combined solution. This ethanol-treated mixture was maintained at −20 °C for 1 h before being centrifuged once more. The collected precipitate was dissolved in sterile water, followed by the addition of 1 mL of 5% phenol solution and 5 mL of concentrated sulfuric acid. After a five-minute equilibration period at ambient temperature, optical density measurements were taken at 490 nm wavelength.

### 2.13. Biofilm Metabolic Activity Assay

The experimental procedure involved combining bacterial cultures (1 × 10^6^ CFU/mL) with equivalent quantities of test compounds in 96-well plates, which were then left to incubate for biofilm development. Following overnight incubation, the liquid above the settled material was carefully removed. Subsequently, each well received identical amounts of MTT reagent (10 mg/mL) and LB nutrient broth, with the plates being kept for an additional 3-h period. After this incubation, the liquid phase was eliminated, and DMSO was introduced to dissolve the purple formazan precipitates. Optical density readings were taken at 570 nm wavelength. The relative bacterial viability was determined through the following calculation: Bacterial viability (%) = Bacterial metabolic activity (%) = (OD570 nm of experimental group/OD570 nm of control group) × 100%.

### 2.14. qPCR

The bacterial culture with a concentration of 1 × 10^6^ CFU/mL was incubated alongside experimental compounds under constant agitation at 37 °C for one day. Following this incubation period, a 1 mL sample was subjected to centrifugation at 8000 rpm for 60 s under refrigerated conditions (4 °C), after which the liquid portion was removed. The remaining cellular material was treated with lysozyme solution and left undisturbed for ten minutes. Next, 1 mL of TransZol Up reagent was introduced to the sample, which was then vigorously mixed for 1 min using a vortex mixer before being kept at ambient temperature for 5 min. The resulting liquid phase was separated, to which 200 μL of chloroform were subsequently introduced.

The sample solution was vigorously mixed using a vortex mixer for 60 s and allowed to settle at room temperature for 25 min. Subsequently, it underwent centrifugation at 12,000× *g* for 15 min while maintaining a temperature of 4 °C. The clear upper layer obtained was then combined with an identical quantity of isopropanol and kept undisturbed for 10 min. Following a second centrifugation procedure, the precipitated material was cleansed with 75% ethanol solution, air-dried, and subsequently dissolved in water treated with diethyl pyrocarbonate (DEPC). The quality of extracted RNA was assessed through electrophoresis on agarose gels, followed by complementary DNA (cDNA) production employing a specialized reverse transcription kit. Specific primer sequences can be found in Table 1. When examining genes associated with biofilm formation (*pelA*, *PslA*, *algD*), GAPDH functioned as the internal control. For analysis of quorum sensing-related genetic markers, 16S rRNA was utilized as the normalization standard. Each quantitative PCR reaction, with a total volume of 10 μL, comprised 5 μL of 2×SGExcel FastSYBR solution, 0.2 μL of forward and reverse primers each, 3.6 μL of nuclease-free distilled water, and 1 μL of cDNA template. The thermal cycling protocol consisted of: primary DNA strand separation at 95 °C for 3 min, succeeded by 45 repetitions of denaturation at 95 °C for 15 s and primer binding/elongation at. The samples were subjected to a temperature of 60 °C for a duration of 45 s. As a negative control, RNase-Free double-distilled water was utilized, and the comparative expression levels of target genes were determined through the 2^−^^ΔΔCt^ calculation approach.

### 2.15. Pyocyanin Detection Assay

Following the experimental protocol established by Vetrivel et al. [28], bacterial cultures with a concentration of 1 × 10^6^ CFU/mL were mixed with testing compounds and maintained at 37 °C with continuous agitation at 220 rpm for 24 h. After centrifugation at 3000 rpm for 10 min, the liquid phase was separated. Chloroform in equal quantity was introduced to the supernatant and vigorously mixed. The blue-green pigment was subsequently isolated from the organic phase and further processed with 0.2 M hydrochloric acid, yielding a rose-colored liquid whose optical density was quantified at 520 nm wavelength.

### 2.16. Elastase Activity Detection Assay

The bacterial culture at a concentration of 1 × 10^6^ CFU/mL was combined with experimental compounds and subjected to continuous agitation overnight. Following this incubation period, a 50 μL of the liquid phase was extracted and combined with 1 mL of Tris-HCl solution (pH 7.2) supplemented with 10 mg of Congo red dye. This combination was vigorously mixed and allowed to react for 18 h before introducing 0.1 mL of EDTA solution. Subsequently, the reaction mixture was cooled in an ice-water mixture, subjected to centrifugation at 10,000× *g* for 5 min, and the optical density of the resulting supernatant was determined at a wavelength of 495 nm.

### 2.17. Lectin Detection Assay

The bacterial culture (1 × 10^6^ CFU/mL) was combined with experimental compounds and maintained at 37 °C under continuous agitation (220 rpm) for an extended period. Following this incubation, the mixture underwent centrifugation at 3000 rpm for a duration of 10 min to separate the liquid portion. The resulting supernatant was carefully gathered for further processing. Subsequent steps were carried out in strict adherence to the guidelines provided with the commercial microbial lectin ELISA assay kit. Specifically, 10 μL of the collected supernatant was blended with 40 μL of buffer solution designed for sample dilution. To this mixture, 100 μL of detection antibody conjugated with horseradish peroxidase was introduced. This combined solution was then subjected to a 60 min incubation period in a temperature-controlled environment. After this incubation phase, the liquid contents were removed, and each well underwent five complete washing cycles using the designated wash buffer. Subsequently, equal volumes (50 μL each) of Substrate A and Substrate B were introduced into the wells, followed by a 15-min dark incubation at 37 °C. The final step involved adding 50 μL of termination solution and recording the optical density at a wavelength of 450 nm.

### 2.18. Metabolite Analysis

#### 2.18.1. Metabolites Extraction Method

Following a 12 h incubation period with bacterial cultures at a concentration of 1 × 10^6^ CFU/mL in the presence of the experimental compound, microbial cells were harvested through centrifugation. The collected specimens underwent three successive rinses using phosphate-buffered saline maintained at low temperatures, with each washing cycle involving a 5 min spin at 5000 rpm under refrigerated conditions (4 °C). After thorough removal of the liquid phase, 300 μL of an 80% methanol-water solution was introduced. This preparation underwent rapid freezing in liquid nitrogen for 5 min, followed by gradual thawing on an ice bath, subsequent vigorous mixing for 30 s, and ultrasonic treatment lasting 6 min. The processed samples were then subjected to another centrifugation step under identical conditions (5000 rpm, 1 min, 4 °C). The resulting supernatant was lyophilized and reconstituted using a 10% methanol solution before being introduced into the liquid chromatography-tandem mass spectrometry instrumentation for detailed examination [29,30].

#### 2.18.2. LC-MS Analysis

The liquid chromatography-mass spectrometry (LC-MS) experiments were conducted using a Vanquish liquid chromatography instrument (Thermo Fisher, Dreieich, Germany) connected to either an Orbitrap Q ExactiveTM HF or Q ExactiveTM HF-X mass analyzer (Thermo Fisher, Dreieich, Germany). Separation was achieved on a Hypersil Gold column (100 × 2.1 mm, 1.9 μm particle size) with a mobile phase flow rate of 0.2 mL/min over a 12 min linear gradient. For both positive and negative ionization modes, the mobile phases consisted of solvent A (aqueous solution containing 0.1% formic acid) and solvent B (methanol). The gradient program was implemented as: initial 2% B maintained for 1.5 min, followed by a linear increase to 85% B over 3 min, then to 100% B by 10 min, returning to 2% B at 10.1 min, and holding at 2% B until 12 min. Mass spectrometric detection was performed in positive/negative switching mode with the following parameters: electrospray voltage at 3.5 kV, capillary maintained at 320 °C, sheath gas flow of 35 psi, auxiliary gas flow of 10 L/min, S-lens RF level at 60, and auxiliary gas heater temperature set to 350 °C.

#### 2.18.3. Data Processing and Metabolite Identification

The LC-MS-derived data files underwent processing through the XCMS platform, which facilitated the alignment of chromatographic peaks, detection of individual peaks, and quantification of metabolites. Subsequently, these processed data were cross-referenced with a comprehensive secondary spectral database, utilizing adduct ion information and maintaining a mass accuracy threshold of 10 ppm, to achieve metabolite characterization. Following the removal of background signals through comparison with blank sample analyses, the initial quantitative measurements were subjected to normalization using a specific mathematical approach: Normalized peak intensities = (Sample measurement value)/(Total sample measurements/Total QC1 measurements).

Substances exhibiting variability exceeding 30% in their relative peak area measurements across quality control samples were excluded from further analysis. This rigorous filtering process yielded definitive metabolite identification outcomes along with their comparative abundance measurements. Data processing was carried out under the Linux CentOS 6.6 operating system, using R 4.2.1 and Python 3.9.0 programming languages. The core analysis of metabolomics employed the R language-specific software packages: MetaboAnalystR (2.0.1) and ropls (1.18.8).

### 2.19. Hemolysis Activity Test Experiment

Collect blood samples from healthy rabbits and separate the plasma by centrifugation at 1000× *g* for 10 min. After discarding the serum, wash the red blood cells with PBS buffer and prepare a 1% cell suspension. Treat the red blood cells with BA, CTX individually or in combination, maintaining the mixture at 37 °C for 60 min before another centrifugation step at 1000× *g* for 10 min. Transfer 100 μL of the resulting supernatant to measure optical density at 570 nm wavelength. Include PBS solution as the negative reference and 0.2% Triton X-100 solution as the positive control. The percentage of hemolysis is determined using the following equation: [(Sample absorbance − PBS absorbance)/(Triton X-100 absorbance − PBS absorbance)] × 100%.

### 2.20. Data Analysis

Metabolite identification was achieved through comprehensive database searches, including the Kyoto Encyclopedia of Genes and Genomes (KEGG), Human Metabolome Database (HMDB), and LIPID Metabolites and Pathways Strategy (LIPIDMAPS) repositories. All experimental procedures were repeated at least three times to ensure reliability. Quantitative data are expressed as arithmetic mean values with accompanying standard deviation values. All data are presented as mean ± standard deviation (mean ± SD), and statistical analysis was performed using GraphPad Prism 9.0 software. Comparisons among multiple groups were conducted using one-way analysis of variance (one-way ANOVA). If the overall difference between groups was statistically significant (*p* < 0.05), Tukey’s multiple comparisons test was used for pairwise comparisons and multiple testing correction was controlled. The threshold for statistical significance was established at three levels: * for *p* < 0.05, ** for *p* < 0.01, and *** for *p* < 0.001.

## 3. Results

### 3.1. Determination of Drug Combination Relationships

The MICs of baicalin (BA) against *Pseudomonas aeruginosa* PAO1 and multidrug-resistant *Pseudomonas aeruginosa* (MRPA) are 5000 μg/mL and 10,000 μg/mL, respectively; while the MICs of cefotaxime (CTX) against these two bacterial strains are 7.8 μg/mL and 15.6 μg/mL, respectively. In addition, the MICs of gentamicin (GM), used as a positive control, against PAO1 and MRPA are 0.78 μg/mL and 1.56 μg/mL in sequence. When 1/32 MIC of BA is combined with CTX, the MIC of CTX against MRPA is reduced by 4-fold, showing a synergistic effect. When 1/2 MIC of BA is combined with CTX, the MIC of CTX against PAO1 is reduced by 2-fold, exhibiting an additive effect. When BA is combined with GM, the antibacterial effects against both MRPA and PAO1 show a partial synergistic effect.

### 3.2. Bactericidal Activity of BA in Combination with CTX

**Figure 1:**
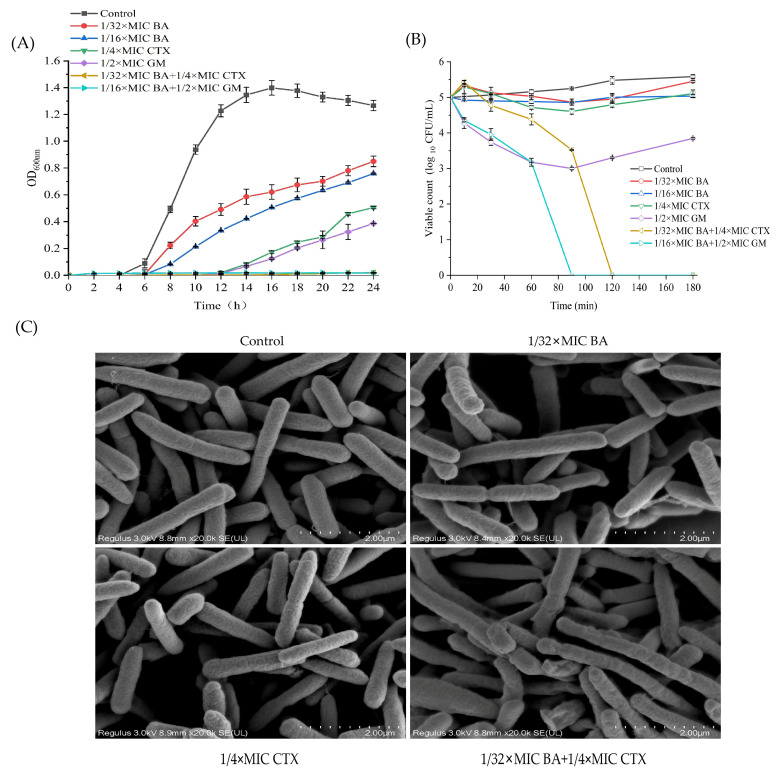
The combined use of BA and antibiotics on the growth curve (**A**) and bactericidal kinetics (**B**) of MRPA; (**C**) The effect of the combined use of BA and CTX on the cell morphology of MRPA as observed under a scanning electron microscope. Error bars represent standard deviations (*n* = 3).

Growth curve assay results showed that when MRPA was treated with 1/32×MIC BA or 1/4×MIC CTX alone, bacterial growth trends appeared at 6 h and 12 h, respectively. However, the combination of 1/32×MIC BA and 1/4×MIC CTX completely inhibited MRPA growth within 24 h (Figure 1A). In addition, the combination of 1/16×MIC BA and 1/2×MIC GM can also achieve complete inhibition of bacterial growth within 24 h (Figure 1A). Subsequently, a bactericidal kinetics assay was performed to further evaluate the effect of the combination of BA and CTX on the bactericidal rate against MRPA. As shown in Figure 1B, the number of untreated MRPA bacteria continued to increase within 180 min. No significant downward trend in colony count was observed within 180 min when 1/4×MIC CTX or 1/32×MIC BA was used alone. In contrast, when 1/32×MIC BA was combined with 1/4×MIC CTX, MRPA was completely eliminated at 120 min. In addition, 1/2×MIC GM alone failed to effectively inhibit bacterial growth within 180 min, while the combination of 1/16×MIC BA and 1/2×MIC GM achieved complete clearance of MRPA at 90 min (Figure 1B). These results indicate that BA enhances the bactericidal activity of CTX and GM. Scanning electron microscopy observations revealed that untreated MRPA cells had intact morphology, smooth surfaces, and clear edges. Treatment with 1/32×MIC BA or 1/4×MIC CTX alone did not cause damage to bacterial morphology. However, when 1/32×MIC BA was combined with 1/4×MIC CTX, MRPA cells exhibited rupture, shrinkage, and leakage of cellular contents (Figure 1C). These findings suggest that the combination of BA and CTX enhances the disruption of MRPA cell membranes, thereby leading to cell death.

### 3.3. Impact of BA Combined with CTX on Bacterial Cell Membrane and Cell Wall Permeability

**Figure 2:**
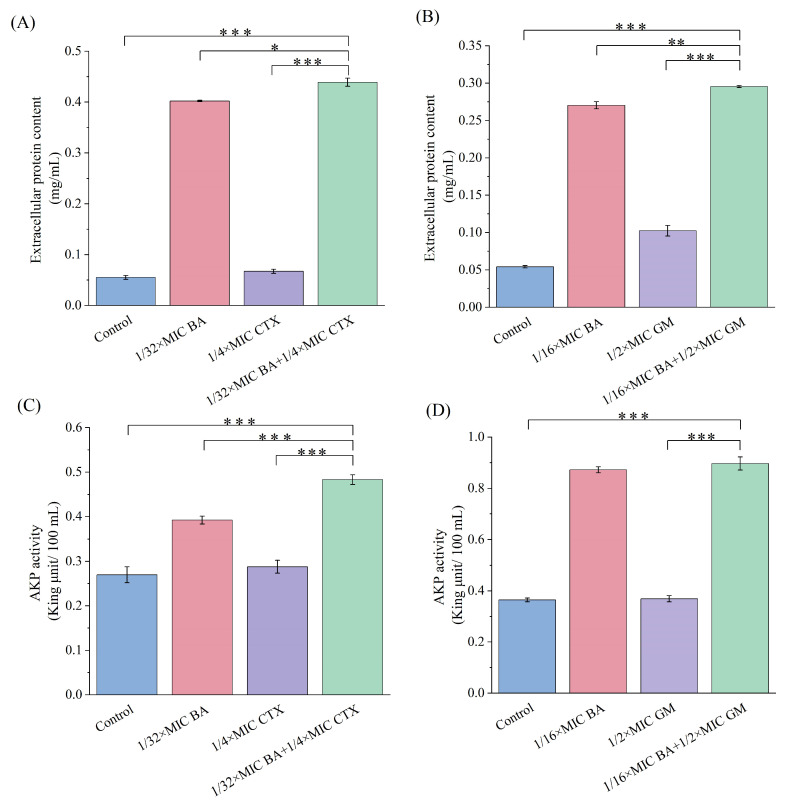
The effect of combining BA with antibiotics on the permeability of the cell membrane (**A**,**B**) and cell wall (**C**,**D**) of MRPA cells. Error bars represent standard deviations (*n* = 3). *** *p* ≤ 0.001, ** *p* < 0.01, * *p* ≤ 0.05.

The combined use of BA and CTX exerts a significant regulatory effect on the permeability of bacterial cell membranes and cell walls, as demonstrated by intracellular protein leakage assays and alkaline phosphatase (AKP) activity detection. In intracellular protein leakage experiments, untreated MRPA shows minimal intracellular protein leakage at only 55 μg/mL. When treated with 1/32×MIC BA alone, the leakage level reaches 422 μg/mL, which is significantly higher than that of 1/4×MIC CTX monotherapy (67 μg/mL). Notably, the combination of 1/32×MIC BA and 1/4×MIC CTX further increases intracellular protein leakage to 439 μg/mL, showing a significant enhancement compared with 1/4×MIC CTX alone (Figure 2A). In terms of AKP activity (Figure 2C), the extracellular AKP activity of untreated MRPA is 0.27 King μnits/100 mL. Treatment with 1/4×MIC CTX alone does not significantly change the extracellular AKP level, which remains similar to that of the control group. However, when 1/4×MIC CTX is combined with 1/32×MIC BA, the extracellular AKP activity of MRPA increases to 0.48 King μnits/100 mL, approximately twice that of the 1/4×MIC CTX monotherapy group (Figure 2C). Since AKP is a key enzyme located in the periplasmic space or cell wall, its increased extracellular release indicates damage to the cell wall structure and increased permeability. These results collectively confirm that BA, when combined with CTX, significantly enhances the permeability of bacterial cell membranes and cell walls, leading to increased leakage of intracellular proteins and extracellular release of AKP. In addition, the combination of 1/16×MIC BA and 1/2×MIC GM similarly enhances the permeability of MRPA’s cell wall and cell membrane (Figure 2B,D).

### 3.4. Molecular Docking Simulation of the Interaction Between BA and WbpE Transaminase

The binding mode and potential binding sites of BA and WbpE transaminase were further explored through molecular docking technology. The three-dimensional structure of BA was described in our previous study [25].

**Figure 3:**
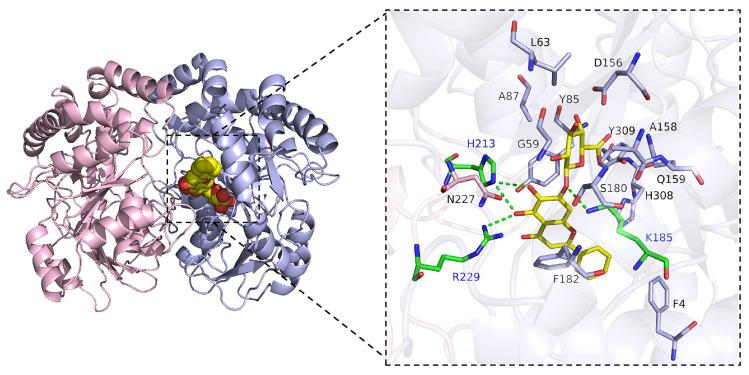
Overview of the top-ranked docking conformations of BA with WbpE transaminase.

Figure 3 presents the best docking model of BA and WbpE transaminase. BA is stably embedded in the cavity region formed by the Leu63, Ala158, Gln159, Tyr85, Lys185, Phe4, His308, Ala87, Phe182, Tyr309, Asp156, Gly59, Ser180, and B chain Asn227, His213, Arg229 of the WbpE transaminase A chain. In the interaction analysis, it was found that this binding is mainly driven by two types of non-covalent forces: first, BA forms stable 4 groups of hydrogen bonds with Lys185(A), His213(B), and Arg229(B), enhancing the specificity and selectivity of the binding; second, the hydrophobic part of BA forms significant hydrophobic interactions with the surrounding 13 residues, effectively eliminating the interference of water molecules. The synergistic effect of these two types of interactions enables BA to form a stable binding in the protein conformation of WbpE transaminase. The prediction results of the AutoDock software show that the binding energy between BA and WbpE transaminase is −10.342 kcal/mol. This confirms the effective binding of BA and the active site of WbpE transaminase, which may be a potential target for enhancing cell wall permeability.

### 3.5. Antibiofilm Activity of BA Combined with CTX Against MRPA

**Figure 4:**
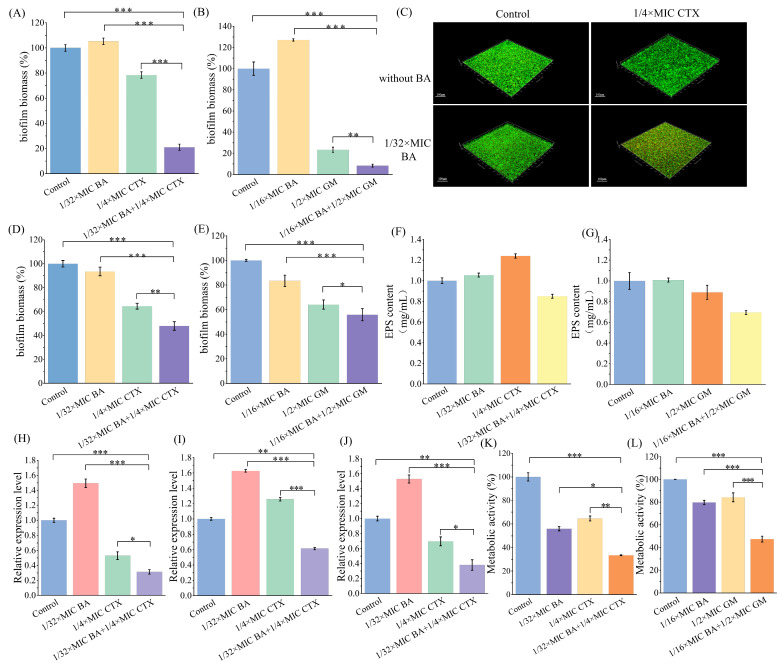
The antibacterial membrane activity of MRPA by the combination of BA and antibiotics. (**A**,**B**) The influence of the combination of BA and antibiotics on the formation of MRPA biofilm; (**C**,**D**) The influence of the combination of BA and antibiotics on the decomposition of MRPA biofilm; (**E**) The influence of the combination of BA and CTX on the biomass of MRPA biofilm, bar = 100 µm; (**F**,**G**) The influence of the combination of BA and antibiotics on the EPS content of MRPA biofilm components; (**H**–**J**) The influence of the combination of BA and CTX on the expression of genes *pslA*, *PelA* and *algD* in MRPA biofilm; (**K**,**L**) The influence of the combination of BA and antibiotics on the cellular metabolic activity of MRPA biofilm. Error bars represent standard deviations (*n* = 3). *** *p* ≤ 0.001, ** *p* < 0.01, * *p* ≤ 0.05.

The antibiofilm activity of the combination of BA and CTX was evaluated using the crystal violet staining method. As shown in Figure 4A, treatment with 1/4×MIC CTX alone resulted in a 22% inhibition rate of MRPA biofilm formation. When combined with 1/32×MIC BA, 1/4×MIC CTX significantly enhanced the inhibition of biofilm formation, achieving an inhibition rate of 79%. In addition, the combination of 1/32×MIC BA and 1/4×MIC CTX also strengthened the degradation effect on preformed 1-day-old MRPA biofilms (Figure 4C). Compared with 1/4×MIC CTX alone, the combination of 1/4×MIC CTX and 1/32×MIC BA increased the degradation rate of 1-day-old biofilms by 16% (Figure 4C). These results indicate that BA effectively enhances the inhibitory effect of CTX on the formation and degradation of MRPA biofilms. Similarly, the combination of 1/16×MIC BA and 1/2×MIC GM also promoted the inhibition and degradation of MRPA biofilms (Figure 4B,D). Laser confocal microscopy observations revealed that after combined treatment with 1/32×MIC BA and 1/4×MIC CTX, red fluorescence (representing dead cells) was significantly enhanced, indicating that the combination could markedly reduce the biomass of MRPA biofilms (Figure 4E). Subsequently, the effects of the combined application of BA and CTX on the content of extracellular polymeric substances (EPS) in biofilms and the expression of related genes were evaluated using the phenol-sulfuric acid method and quantitative real-time polymerase chain reaction (qPCR). The results showed that, compared with the control group, treatment with 1/32×MIC BA or 1/4×MIC CTX alone failed to reduce EPS content, whereas the combination of 1/32×MIC BA and 1/4×MIC CTX decreased MRPA EPS by 15% (Figure 4F). However, 1/2×MIC GM alone reduced MRPA EPS content by 12%, and when combined with 1/16×MIC BA, the EPS content was reduced by 31% (Figure 4G). The combination of BA and CTX was also confirmed to enhance the inhibition of MRPA biofilm-related genes *pslA*, *pelA*, and *algD* (Figure 4H–J). The results showed that, compared with 1/4×MIC CTX alone, the combination with 1/32×MIC BA led to more significant downregulation of *pslA*, *pelA*, and *algD* gene expression, with reductions of 69%, 39%, and 62%, respectively (Figure 4H–J). Furthermore, the metabolic activity of MRPA biofilm cells after combined treatment with BA and CTX was detected using the MTT assay. Compared with the control group, treatment with 1/32×MIC BA or 1/4×MIC CTX alone reduced the metabolic activity of MRPA biofilm cells by 45% and 36%, respectively (Figure 4K). When 1/32×MIC BA was combined with 1/4×MIC CTX, the metabolic activity of MRPA biofilm cells was reduced by 67% (Figure 4K). This significant reduction in metabolic activity indicates lower viability of MRPA under the combined treatment of BA and CTX. In addition, when GM was combined with BA, the metabolic activity was reduced by 52%, 32%, and 36% compared with the control group, BA alone, and GM alone, respectively (Figure 4L).

### 3.6. Impact of BA Combined with CTX on Virulence Factors and Quorum Sensing Genes in MRPA

**Figure 5:**
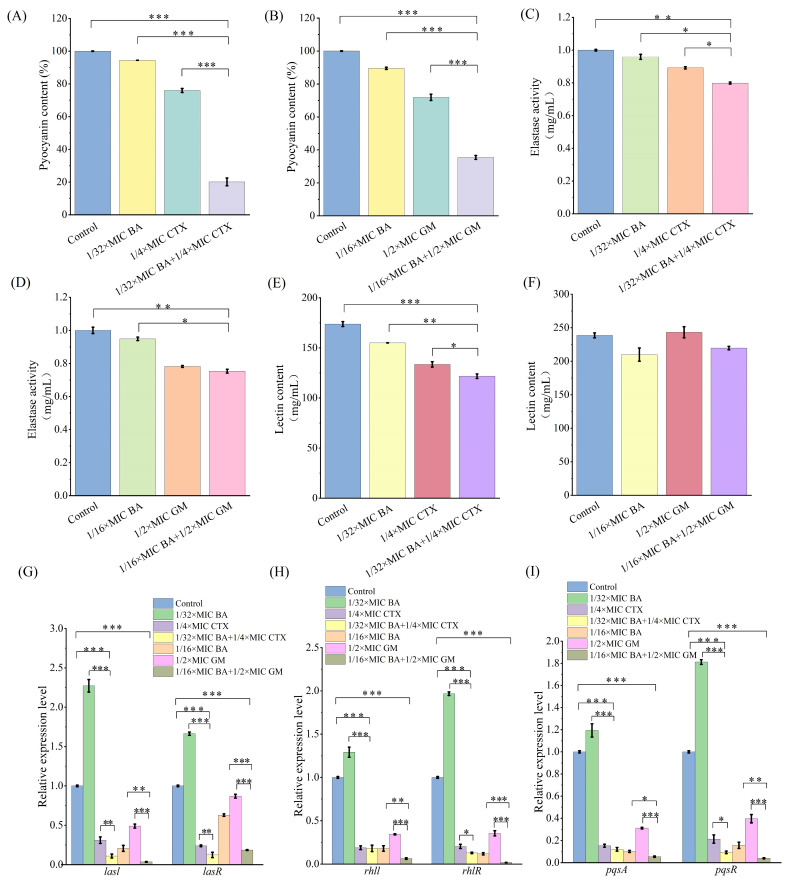
The effects of combining baicalin with antibiotics on the synthesis of virulence factors and quorum sensing genes in MRPA. (**A**,**B**) The influence of combining baicalin with antibiotics on the content of pyocyanin in MRPA; (**C**,**D**) The influence of combining baicalin with antibiotics on the activity of elastase in MRPA; (**E**,**F**) The influence of combining baicalin with antibiotics on the content of agglutinin in MRPA; (**G**) The influence of combining baicalin with cefotaxime on the expression of quorum sensing genes *lasl* and *lasR* in MRPA; (**H**) The influence of combining baicalin with cefotaxime on the expression of quorum sensing genes *rhll* and *rhlR* in MRPA; (**I**) The influence of combining baicalin with cefotaxime on the expression of quorum sensing genes *pqsA* and *pqsR* in MRPA. Error bars represent standard deviations (*n* = 3). *** *p* ≤ 0.001, ** *p* ≤ 0.01, * *p* ≤ 0.05.

*Pseudomonas aeruginosa* can produce and/or secrete a variety of virulence factors, which not only serve as key mediators for bacterial adhesion, motility, and subsequent infection processes but also have their activities precisely regulated through a hierarchical control mode via the quorum sensing (QS) system. Based on this, the present study evaluated the effect of BA combined with CTX on the production of QS-regulated virulence factors in MRPA. The results showed that, compared with the control group and the 1/4×MIC CTX group, the combination of 1/32×MIC BA and 1/4×MIC CTX reduced pyocyanin production by 80% and 74%, respectively (Figure 5A). Similarly, this combined treatment regimen exerted a significant inhibitory effect on the synthesis of elastase and lectin: compared with the control group, their contents decreased by 20% and 30%, respectively; compared with the 1/4×MIC CTX monotherapy group, the reductions were 10.5% and 8.8%, respectively (Figure 5C,E). In addition, the combination of 1/16×MIC BA and 1/2×MIC GM also decreased the levels of virulence factors, with reductions of 65%, 25%, and 8% in pyocyanin, elastase, and lectin, respectively, compared with the control group (Figure 5B,D,F). These results indicate that the combination of BA with antibiotics can effectively inhibit the synthesis of virulence factors in MRPA. Previous studies have confirmed that the QS system plays a crucial role in biofilm formation, bacterial virulence factor expression, and the development of drug resistance [31]. The present study further revealed that the expression of QS-regulated virulence factor-related genes in MRPA was significantly downregulated after combined treatment with BA and CTX (Figure 5G–I). Specifically, compared with the control group, the combination of 1/32×MIC BA and 1/4×MIC CTX significantly downregulated the expression levels of *lasI* and *lasR* genes by 89.03% and 87.62%, respectively (Figure 5G); it also reduced the expression levels of *rhlI* and *rhlR* genes by 81.87% and 87.07%, respectively (Figure 5H); furthermore, the expression of *pqsA* and *pqsR* genes was also significantly inhibited, with downregulation rates reaching 87.9% and 90.6%, respectively (Figure 5I). Notably, the combination of BA and GM also significantly downregulated the expression levels of the aforementioned QS genes (Figure 5G–I).

### 3.7. Molecular Docking Simulation of the Interaction Between BA and LasR/RhlR

**Figure 6:**
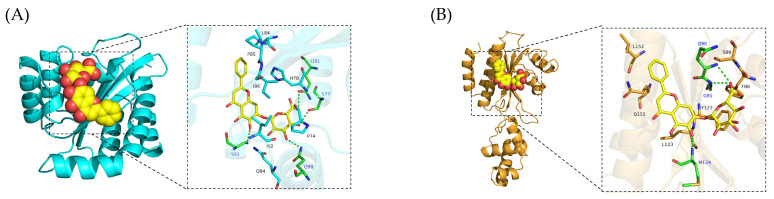
Overview of the top-ranked docking conformations of BA with LasR (**A**) and RhlR (**B**).

The binding mode and potential binding sites of BA and the quorum sensing protein LasR/RhlR were investigated through molecular docking. Figure 6A,B presented the best docking models of BA with LasR and RhlR, respectively. The results showed that BA could stably fit into the cavity region formed by the amino acids Gln98, Pro74, Ile92, Ser91, Ile86, Leu84, Pro85, His78, Gln81, Ser77 and Gln940 of LasR (Figure 6A). Further interaction analysis indicated that BA formed 4 stable hydrogen bond interactions with the amino acids Gln98, Ser77 and Ser91 of the LasR protein. This interaction helped to enhance the selectivity and specificity of the binding. Moreover, the hydrophobic domain of BA interacted significantly with the 7 amino acids Pro74, Leu84, Pro85, etc., adjacent to Gln90, Gly91, Ser89, Ser91, Leu152, Leu123, Met124, Gln155 and Tyr122, thereby effectively reducing the interference of water molecules (Figure 6A).

In the optimal molecular docking conformation of BA with RhlR protein, it could be observed that BA was located in a relatively closed binding site within the RhlR protein. This site was composed of amino acid residues such as Phe88, Gln90, Gly91, Ser89, Ser91, Leu152, Leu123, Met124, Gln155 and Tyr122 (Figure 6B). The interaction analysis of the binding interface indicated that the interaction between BA and the RhlR protein was mainly non-covalent. BA could establish 3 hydrogen bond connections with Gln90, Gly91 and Met124 residues respectively. These directional interactions improved the stability of molecular recognition to a certain extent (Figure 6B). At the same time, the hydrophobic structural region of the BA molecule skeleton formed close hydrophobic contacts with multiple residues such as Ser89, Leu152, Gln155, etc., which was conducive to repelling water molecules within the binding site and further stabilizing the complex structure. The coexistence of these various interactions enabled BA to maintain a relatively stable binding state in the binding pocket of RhlR protein. According to the prediction of the Autodock software, the results show that the binding energy between BA and the LasR protein is −9.373 kcal/mol, and the binding energy between BA and the RhlR protein is −7.681 kcal/mol. This indicates that BA can stably bind to the LasR/RhlR proteins. In contrast, the binding energy between BA and the LasR protein is smaller, which means that the binding structure of BA to LasR is more stable and has a higher affinity.

### 3.8. Metabolomic Changes Induced by BA and CTX in Combination

**Figure 7:**
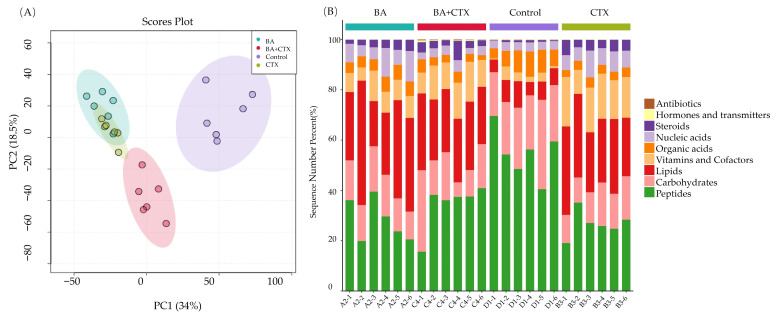
The combined effect of BA and CTX on the overall metabolism of MRPA. (**A**) Principal component analysis. (**B**) Bar chart accumulation.

To further explore the antibacterial mechanism of BA and CTX against MRPA, LC-MS was used to analyze the differences between the control group and drug-treated groups. As shown in Figure 7A, under positive and negative ion modes, distinct sample differences were observed between different treatment groups (BA, BA + CTX, CTX) and the control group, especially in the BA + CTX group, indicating that the combined medication may induce unique metabolic changes (Figure 7A). The stacked bar chart shows the percentage distribution of top-ranked features among different groups (BA, BA + CTX, Control, and CTX). Notably, peptides accounted for a higher proportion in the control group, while lipids were more abundant in the BA and BA + CTX groups. In contrast, carbohydrates significantly increased in the CTX group (Figure 7B). Overall, the distribution patterns of these specific features—such as peptides, lipids, carbohydrates, organic acids, vitamins, and cofactors—varied significantly among the groups.

### 3.9. Identification of Differential Metabolites

**Figure 8:**
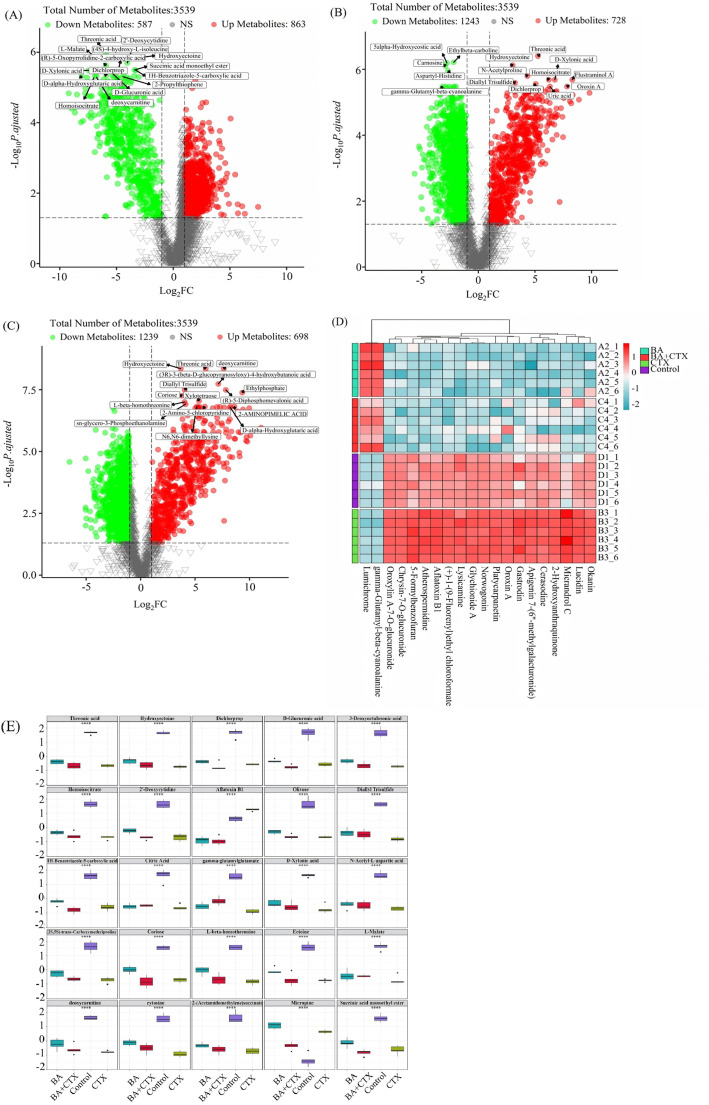
Analysis of significant differential metabolites resulting from the combination of BA and CTX. (**A**) Volcano plot (Control vs. BA + CTX); (**B**) Volcano plot (Control vs. BA); (**C**) Volcano plot (Control vs. CTX); (**D**) Cluster heatmap of differential metabolites; (**E**) Boxplot matrix of differential metabolites. All subfigures are marked with ****, indicating that the differences in the corresponding metabolites between groups are extremely statistically significant (*p* < 0.0001).

In untargeted metabolomics analysis, a total of 3539 metabolites were identified. The volcano plot showed significant differences in metabolite expression between the control group and the BA + CTX group (Figure 8A). Specifically, 863 metabolites were up-regulated and 587 were down-regulated (p.adjusted < 0.05 and |log_2_FC| ≥ 1). Notably, several key metabolites, including threonic acid, L-malate, and 2′-deoxycytidine, exhibited highly significant differential expression (Figure 8A). There were more significant differences in the expression of metabolites between the control group and the BA group (Figure 8B) or the CTX group (Figure 8C). Specifically, 728 and 698 metabolites were upregulated, while 1243 and 1239 metabolites were downregulated. The hierarchical clustering heatmap of differential metabolites (VIP > 1.0 and *p* < 0.05) displayed differences in metabolite abundance among the four groups (BA, BA + CTX, Control, and CTX) (Figure 8D). Key metabolites such as Okanin and 2-Hydroxyanthraquinone were highly abundant in the Control group (Figure 8D). In the BA group, the abundance of some key metabolites decreased, forming a significant difference from the control group, indicating the interference of BA monotherapy on the metabolic profile (Figure 8D). The treatment with CTX alone can significantly induce the accumulation of a large number of key metabolites (Figure 8D). In the BA + CTX group, key metabolites were predominantly blue-green, completely separated from the red region of the Control group, showing the significant metabolic reprogramming, with the abundance of most key metabolites strongly inhibited (Figure 8D). This result indicates that both the BA and CTX single treatment groups can significantly alter the metabolic profile. Among them, the use of CTX alone can strongly induce the enrichment of key metabolites, while the use of BA alone interferes with metabolism and reduces the levels of some key metabolites. When BA and CTX are used together, it is not a simple addition but rather produces a strong synergistic regulatory effect, which can reverse the abnormal accumulation of metabolites caused by CTX and cause the most significant reprogramming of the overall metabolic profile, manifested as the significant inhibition of most key metabolites.

The matrix of differential metabolite boxplots further clearly demonstrated differences in metabolite contents among different groups (*p* < 0.05) (Figure 8E). The median metabolite content in the BA + CTX group was often different from that in the BA, Control, and CTX groups. Metabolites marked with *** (*p* < 0.001), such as coriose, gamma-glutamylglutamate, 1H-benzimidazole-5-carboxylic acid, D-xylonic acid, and L-malate, reflected the most unique effect of the combined treatment (BA + CTX) on metabolites (Figure 8E). The boxplots of the BA and CTX groups differed significantly from those of the BA + CTX group, indicating differences in metabolic regulatory pathways between monotherapy and combined treatment (Figure 8E). Based on the structure and function of metabolites, we found that metabolites such as D-gluconic acid, olivose, coriose, and D-xylonic acid belong to the carbohydrate/sugar metabolism category (Figure 8E). Among them, D-gluconic acid is involved in the synthesis of certain metabolites related to cell wall formation, which is crucial for maintaining the structural integrity of the bacterial cell wall [32]. Those involved in central carbon metabolism provide energy for bacterial life activities and can reflect the energy metabolism status or stress response of bacteria [33,34]. Metabolites including gamma-glutamylglutamate, N-acetyl-L-aspartic acid, and 1H-benzimidazole-5-carboxylic acid belong to the amino acid and derivative category (Figure 8E). Among them, gamma-glutamylglutamate and N-acetyl-L-aspartic acid are indirectly associated with protein synthesis through participation in the regulation of amino acid metabolic homeostasis and play a role in cell signal transduction [35,36,37]. Metabolites such as succinic acid monomethyl ester and diallyl trisulfide belong to the lipid and derivative category (Figure 8E). They can indirectly participate in the regulation of bacterial cell membrane function and adaptation to the living environment by participating in signal molecule synthesis and exerting antibacterial activity, with some components playing an auxiliary role in maintaining the stability of membrane-related physiological processes [38,39]. Cytosine belongs to the nucleotide and derivative category and is a typical nucleotide precursor (Figure 8E). Antibacterial substances such as micropline are defensive secondary metabolites synthesized by microorganisms, helping them inhibit the growth of other microorganisms in survival competition [40]. In addition, organic acid metabolites such as citric acid and threonic acid, as key intermediates in core energy metabolism pathways (e.g., tricarboxylic acid cycle, glycolysis-derived pathways), their content changes can directly reflect dynamic adjustments in metabolic flux, providing important insights for analyzing bacterial energy metabolism status [41].

### 3.10. Enrichment Analysis of Metabolic Pathways in MRPA Induced by the Combination of BA and CTX

**Figure 9:**
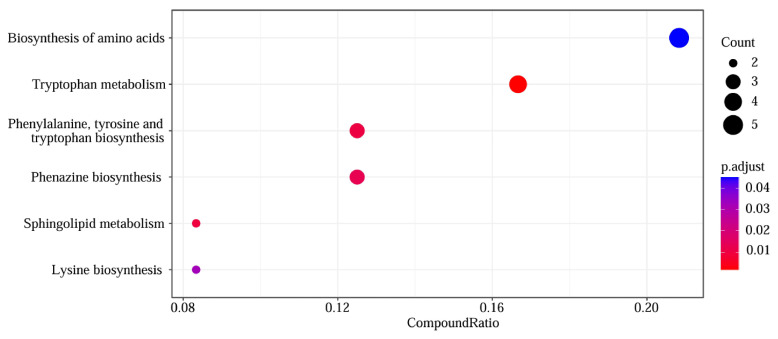
KEGG pathway enrichment analysis of differential metabolites.

ORA revealed six significantly enriched KEGG pathways (Figure 9). The most enriched pathway was Biosynthesis of amino acids (CompoundRatio = 0.20, Count = 5, p.adjust < 0.01), followed by Tryptophan metabolism (CompoundRatio = 0.16, Count = 4, p.adjust ≈ 0.04). Pathways related to amino acid synthesis (e.g., Phenylalanine, tyrosine and tryptophan biosynthesis) and lipid metabolism (e.g., Sphingolipid metabolism) also showed significant enrichment (CompoundRatio range: 0.08–0.12; Count range: 2–3; p.adjust ≤ 0.04).

### 3.11. Safety Analysis

**Figure 10:**
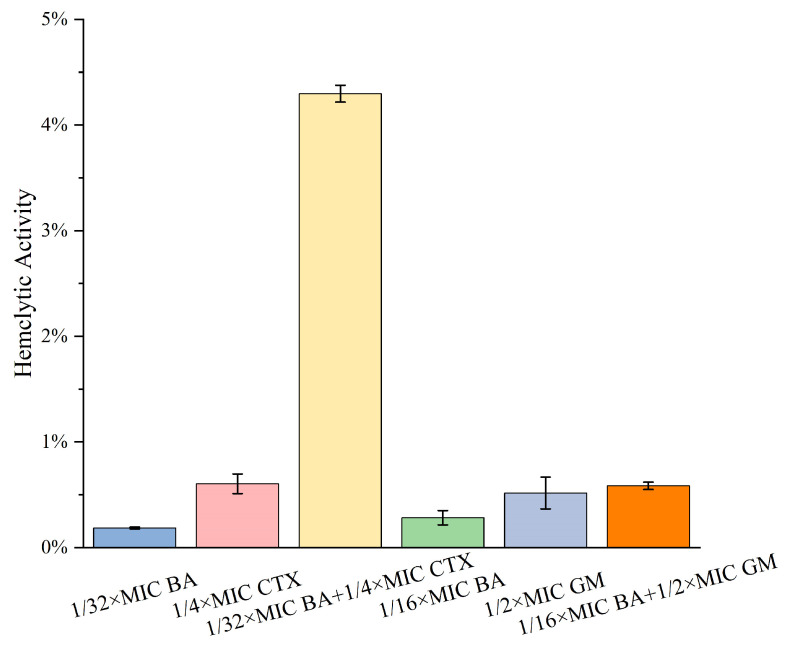
Analysis of hemolytic activity when BA is combined with antibiotics.

Whether it is the treatment of red blood cells with BA alone or with CTX alone, or in combination, the hemolytic activity is all below 5% (Figure 10), indicating that these drugs have a relatively low direct damage effect on red blood cells, and do not cause obvious rupture of red blood cells or release of hemoglobin, showing good biological safety.

## 4. Discussion

*Pseudomonas aeruginosa* has attracted growing concern among clinicians due to its high mortality and drug resistance rates. The combination of natural plant extracts with antibiotics is expected to become a new option for clinical treatment. Therefore, this study systematically evaluated the antibacterial activity and mechanism of action of the combination of baicalin (BA) and cefotaxime (CTX). The results showed that the combination of BA and CTX significantly enhanced antibacterial activity through multi-target effects. Specifically, the combination of BA and CTX exhibited synergistic anti-multidrug-resistant *Pseudomonas aeruginosa* (MRPA) activity, accelerated the bactericidal rate, and disrupted bacterial morphology (Figure 1). Bacterial cell membranes and cell walls serve as the first line of defense against antibiotics, and decreased permeability is one of the core mechanisms underlying MRPA resistance to CTX [42]. In this study, the combination of 1/32×MIC BA and 1/4×MIC CTX significantly increased intracellular protein leakage in MRPA, with an increase of 372–384 μg/mL compared to the 1/4×MIC CTX alone group (Figure 2). Meanwhile, the AKP activity of the combined treatment group was twice as high as that of the 1/4×MIC CTX single treatment group (Figure 2). The molecular docking results further revealed that BA could bind to the WbpE transaminase related to LPS assembly, suggesting that BA might inhibit LPS synthesis by interacting with WbpE transaminase, thereby enhancing the destructive effect of CTX on the cell wall of MRPA cells (Figure 3). This result is consistent with the findings of Abdullah et al., who discovered that the natural source antimicrobial peptide Thanatin and its derivatives directly bind to and inhibit the periplasmic protein LptA_m_ of the LPS transport complex, blocking the transport of LPS from the inner membrane to the outer membrane, inhibiting the biosynthesis of the outer membrane, and thereby enhancing antibiotic sensitivity [43]. Bacterial lipid metabolism and lipidome remodeling directly determine the outer membrane permeability by regulating membrane fluidity, density, surface charge and structural integrity, thereby controlling antibiotic penetration and antibiotic resistance phenotypes [44]. Metabolomic analysis revealed that the combination of BA and CTX significantly downregulated metabolites such as Succinic acid monomethyl ester and Diallyl trisulfide (Figure 8C). The function of these lipid metabolites (regulating bacterial membrane integrity) is consistent with the enrichment of lipid metabolism pathways (e.g., sphingolipid metabolism) in over-representation analysis (ORA) (Figure 9). Thus, BA may perturb lipid metabolism-related pathways to increase membrane fluidity or disrupt cell wall structures, creating conditions for CTX to enter bacterial cells. Biofilm formation is another core reason for enhanced MRPA resistance. The physical barrier effect and metabolic heterogeneity of EPS can significantly reduce antibiotic sensitivity [45]. This study found that the inhibition rate of MRPA biofilm formation by the combination of 1/32×MIC BA and 1/4×MIC CTX was 57% higher than that of the 1/4×MIC CTX alone group, and the degradation rate of 1-day-old mature biofilms was increased by 16% (Figure 4A,C). Previous studies have shown that the structural stability of *Pseudomonas aeruginosa* biofilms is highly dependent on the synthesis of Psl, Pel, and alginate [46]. Inhibiting the expression of these polysaccharide synthesis genes can directly disintegrate the biofilm scaffold [46]. This study found that the combined group may disrupt the structural integrity by inhibiting the expression of *pslA*, *pelA*, and *algD* genes (Figure 4H–J). In addition, MTT assay confirmed that the combination of 1/32×MIC BA and 1/4×MIC CTX reduced the proportion of metabolically active cells in biofilms by 67%, which was much higher than that of 1/32×MIC BA alone (45%) or 1/4×MIC CTX alone (36%) (Figure 4K). The metabolomics results indicate that the number of differentially expressed metabolites between the control group and the BA group, as well as the CTX group, is greater than that between the combined group (Figure 8A–C). This suggests that single drug treatment can cause extensive non-specific perturbations in the metabolic profile of the organism. Although the total number of differentially expressed metabolites in the BA + CTX combined treatment is relatively reduced, it shows significant and directional regulatory effects on key metabolites, such as down-regulating Citric Acid, Threonic acid, and L-Malate in the tricarboxylic acid cycle and sugar metabolism-derived pathways (Figure 8D,E). This indicates that the combined medication achieves a more potent metabolic reprogramming through a synergistic effect rather than directly exacerbating metabolic disorders. These findings indicate that the combination group not only physically disrupts biofilms but also impairs bacterial viability by inhibiting metabolites related to sugar metabolism and energy metabolism. These results are in agreement with the view that antibiotic resistance mediated by bacterial biofilms is highly dependent on the metabolic reprogramming within the bacterial community [47]. The pathogenicity of *Pseudomonas aeruginosa* depends on the secretion of virulence factors such as pyocyanin and elastase, and the expression of these factors is tightly regulated by the quorum sensing (QS) system. The QS system of *Pseudomonas aeruginosa* mainly consists of three components: las, rhl and pqs [48]. These systems work together to regulate the secretion of virulence factors and the oxidative stress response pathway of the bacteria, significantly influencing the drug resistance and pathogenicity of the bacteria [48]. Compared with the control group, the combination of 1/32×MIC BA and 1/4×MIC CTX reduced pyocyanin production by 80% and decreased the levels of elastase (20%) and lectin (30%), indicating that the combination group not only inhibits bacterial growth but also reduces pathogenicity by attenuating virulence factor synthesis (Figure 5A,C,E). Furthermore, our study found that the combination of 1/32×MIC BA and 1/4×MIC CTX significantly inhibited the expression of QS genes (*lasI*, *lasR*, *rhlI*, *rhlR*, *pqsA* and *pqsR*) (Figure 5G–I). The molecular docking results also revealed that BA could stably bind to lasR/rhlR proteins (Figure 6). The above results are highly consistent with those reported by Chen et al., which indicated that the natural small molecule alkaloid Harmine can simultaneously bind to the three key QS receptors (LasR, RhlR, and PqsR), thereby inhibiting various virulence phenotypes such as the secretion of pyocyanin and extracellular proteases [49]. This suggests that BA can act as a QS inhibitor and work synergistically with CTX to exert bactericidal effects. Metabolic reprogramming is an important strategy for bacteria to adapt to external stress and maintain drug resistance. Through untargeted metabolomic analysis, this study revealed that the combination of BA and CTX induced significant metabolic disturbance in MRPA, with a total of 863 upregulated and 587 downregulated metabolites (Figure 8A). The intensity and range of regulation were significantly greater than those in the BA alone or CTX alone groups (Figure 8D). These differential metabolites were mainly enriched in amino acid metabolism, lipid metabolism (sphingolipid metabolism), and secondary metabolite biosynthesis pathways (Figure 9). In conclusion, BA and CTX synergistically resist MRPA by destroying membrane barriers, inhibiting biofilms, blocking quorum sensing, and interfering with core metabolism, which provides a potential strategy for the treatment of multi-drug-resistant bacterial infections with natural products combined with antibiotics. However, this study still has certain limitations. For instance, no in vivo efficacy verification was conducted, and the clinical conversion dose needs further evaluation. In the future, corresponding animal models can be established to conduct in vivo pharmacodynamics and pharmacokinetic studies, systematically optimize the administration plan and safe dose range, and provide more sufficient experimental basis for the subsequent clinical trial design and clinical application.

## 5. Conclusions

This study reveals the antibacterial and antibiofilm potential of the combined use of BA and CTX against MRPA The results show that the combination of BA and CTX can synergistically inhibit the growth of MRPA. Further investigations demonstrated that the combination of BA and CTX enhances the permeability of bacterial cell membranes/walls and inhibits the synthesis of biofilms and virulence factors by interfering with MRPA’s amino acid metabolism, lipid metabolism, and secondary metabolite biosynthesis pathways. In addition, the combined use of BA and CTX exerts antibiofilm and antivirulence factor activities by downregulating the expression of QS genes. In summary, the combination of BA and CTX represents an effective antibacterial strategy. Delving deeper into the antibiofilm mechanisms driving the synergistic effects of their combination will advance our understanding of the pathogenicity of *Pseudomonas aeruginosa* and offer novel perspectives for managing chronic illnesses linked to biofilm-producing pathogens.

## Figures and Tables

**Table 1:** Primer Sequence Information.

Gene Name	Forward Primer	Reverse Primer
*GAPDH*	CCTCAAGGACTCCGAGAAAGA	AGTTGTACGGCCCCATGC
*16S rRNA*	GCGCAACCCTTGTCCTTAGTT	TGTCACCGGCAGTCTCCTTAG
*PsIA*	AGTCGGTAGATAGCCTGTCGC	GCTGAAGATATCGTCGGAGTAGA
*pelA*	TACGCGCCGATCATCAAC	CTCATCCACAGCGACAACG
*algD*	CTTTGGTTTGGGCTATGTCG	CCGACGCAGATGAACGATAC
*lasl*	CGCACATCTGGGAACTCA	CGGCACGGATCATCATCT
*lasR*	CTGTGGATGCTCAAGGACTAC	AACTGGTCTTGCCGATGG
*rhll*	GTAGCGGGTTTGCGGATG	CGGCATCAGGTCTTCATCG
*rhlR*	GCCAGCGTCTTGTTCGG	CGGTCTGCCTGAGCCATC
*pqsA*	GACCGGCTGTATTCGATTC	GCTGAACCAGGGAAAGAA
*pqsR*	CTGATCTGCCGGTAATTGG	ATCGACGAGGAACTGAAGA

## Data Availability

Data is contained in the paper.

## Abbreviations

AKP	Alkaline Phosphatase
BA	baicalin
CLSM	Confocal laser scanning microscopy
CTX	cefotaxime
DMSO	dimethyl sulfoxide
eDNA	extracellular DNA
EDTA	Ethylene Diamine Tetraacetic Acid
EPS	extracellular polymeric substance
FICI	fractional inhibitory concentration index
GM	gentamicin
HRP	horseradish peroxidase
MIC	Minimum inhibitory concentration
MRPA	Multidrug-resistant *Pseudomonas aeruginosa*
MTT	3-[4,5-dimethylthiazol-2-yl]-2,5-diphenyltetrazolium bromide
PBPs	penicillin-binding proteins
PBS	phosphate-buffered saline
qPCR	quantitative real-time polymerase chain reaction
QS	quorum sensing
SD	standard deviation
